# The Discrepancy between Patient and Clinician Reported Function in Extremity Bone Metastases

**DOI:** 10.1155/2016/1014248

**Published:** 2016-09-20

**Authors:** Stein J. Janssen, Eva A. J. van Rein, Nuno Rui Paulino Pereira, Kevin A. Raskin, Marco L. Ferrone, Francis J. Hornicek, Santiago A. Lozano-Calderon, Joseph H. Schwab

**Affiliations:** ^1^Department of Orthopaedic Surgery, Orthopaedic Oncology Service, Massachusetts General Hospital, Harvard Medical School, Boston, MA, USA; ^2^Department of Orthopaedic Surgery, Orthopaedic Oncology Service, Brigham and Women's Hospital, Harvard Medical School, Boston, MA, USA

## Abstract

*Background*. The Musculoskeletal Tumor Society (MSTS) scoring system measures function and is commonly used but criticized because it was developed to be completed by the clinician and not by the patient. We therefore evaluated if there is a difference between patient and clinician reported function using the MSTS score.* Methods*. 128 patients with bone metastasis of the lower (*n* = 100) and upper (*n* = 28) extremity completed the MSTS score. The MSTS score consists of six domains, scored on a 0 to 5 scale and transformed into an overall score ranging from 0 to 100% with a higher score indicating better function. The MSTS score was also derived from clinicians' reports in the medical record.* Results*. The median age was 63 years (interquartile range [IQR]: 55–71) and the study included 74 (58%) women. We found that the clinicians' MSTS score (median: 65, IQR: 49–83) overestimated the function as compared to the patient perceived score (median: 57, IQR: 40–70) by 8 points (*p* < 0.001).* Conclusion*. Clinician reports overestimate function as compared to the patient perceived score. This is important for acknowledging when informing patients about the expected outcome of treatment and for understanding patients' perceptions.

## 1. Introduction

Treatment for bone metastatic disease is often palliative and aims to maintain function and quality of life for the remaining life span [1, 2]. Traditionally, studies focused on oncological and surgical outcomes (e.g., survival and local recurrence), but more emphasis has been placed on measuring impairment and disability over the past decades [1, 3–5]. The Musculoskeletal Tumor Society (MSTS) recognized this and developed a system—the MSTS score—to evaluate function in patients with musculoskeletal tumors [3]. The validity and reliability of this tool were found to be acceptable when applied to a sample of patients with malignant musculoskeletal tumors [6]. The scoring system has been criticized because it was developed to be completed by a clinician, instead of measuring function as perceived by the patient [1, 7]; however, the MSTS score is still used because of its simplicity and brevity (it consists of six items) [8, 9]. Studies in other fields have demonstrated discrepancies between patient and physician assessment of physical and mental health [10–13]. It is unclear whether the clinician derived MSTS score is representative of the patients' perceived function. We therefore sought to evaluate if there is a difference between patient and clinician reported physical function using the MSTS score in a cohort of patients with bone metastases of the extremities. Secondarily, we compared MSTS domain scores and assessed agreement between the clinician and patient perceived scores.

## 2. Materials and Methods

### 2.1. Study Design

Our institutional review board approved secondary use of prospectively collected data for the purpose of this study, and a waiver of informed consent was obtained. We included data from the first 128 patients who completed a set of physical function questionnaires for two prior studies. These studies compared physical function questionnaires in patients with lower (*n* = 100) and upper (*n* = 28) extremity bone metastases, myeloma, or lymphoma [14]. Only English-speaking patients aged 18 years or above who were able to provide informed consent were approached for these studies. Patients were enrolled between June 2014 and September 2015 from two orthopaedic oncology clinics. Patients were included regardless of previous treatment and disease stage [14]. Seventeen patients declined participation for the initial study, and three patients were excluded because of incomplete questionnaires.

An ante hoc sample size calculation determined that we would need a minimum of 128 patients to find an effect size of 0.25 with an alpha of 0.05 and power of 0.80 using a paired *t*-test comparing the clinician reported MSTS score with the patient perceived MSTS score.

### 2.2. Outcome Measures

Our primary outcome measure was the Musculoskeletal Tumor Society (MSTS) score, introduced in 1983 and modified in 1993 [3]. This scoring system was developed to be completed by a clinician—physician or physician extender—and it aims to assess physical function in patients with lower and upper extremity tumors. The modified version (1993) of the MSTS score consists of six domains, each scored on a scale from 0 to 5, with a higher score indicating better function. The total score, ranging from 0 to 30, can be transformed to a point scale of 0 to 100. There are two versions: one for lower extremity tumors and one for upper extremity tumors. These versions have three domains in common, pain, function, and emotional acceptance, and three region specific domains. The region specific domains for the lower extremity are use of supports, walking ability, and gait. The region specific domains for the upper extremity are hand-positioning, dexterity, and lifting ability. Patients completed one of the two versions based on the location of their most disabling bone metastasis.

In addition, patients completed questions about their level of education, marital status, presence of other disabling conditions, prior treatment, and other bone or visceral metastases. Prior treatment and presence of other metastases were also derived from medical records. We extracted age, sex, race, and location of bone metastasis from the medical records.

Two research fellows (SJ and EvR)—blinded to the patients' answers—independently completed the MSTS score based on the clinicians' report in the medical record of the patient; we used the report that was written at the time (or within a few days) of survey completion by the patient. Reports completed by the orthopaedic oncologist, medical oncologist, and physical therapist were used to complete the MSTS score. We averaged the scores assigned by the two researchers per domain and for the overall MSTS score. To assess reliability of extracting this data from medical records, we assessed difference in overall MSTS score and domain scores between researchers and assessed their interobserver agreement.

### 2.3. Statistical Analysis

We used frequencies with percentages to describe categorical variables and median with interquartile range for continuous variables as histograms suggested nonnormality.

The nonparametric Wilcoxon signed rank test was used to assess the difference between patient and clinician domain scores and overall MSTS scores as data was not normally distributed.

We assessed the relationship between the patient and clinician MSTS and domain scores using both Spearman rank correlation and intraclass correlation (ICC). Spearman rank correlation determines the relationship between two variables (range: −1 to 1): a score of 1 indicates a perfect correlation, 0 indicates no correlation, and −1 indicates a perfect inverse correlation. We used bootstrapping (number of resamples: 1,000) to calculate *p* values and 95% confidence intervals for the Spearman rank correlation coefficients. The intraclass correlation coefficient also assesses a relationship between two variables but accounts for discrepancy in measurements and therefore measures absolute agreement. We calculated the ICC through a two-way mixed-effects model with absolute agreement for the overall MSTS score and the domain scores. As with the Spearman rank correlation coefficient, an ICC of 1 reflects perfect agreement, whereas 0 reflects no agreement.

Additionally, we assessed difference in domain and total scores between the two researchers using the Wilcoxon signed rank test and assessed their interobserver agreement per domain and overall score using the ICC.

### 2.4. Patient Characteristics

The median age was 63 years (interquartile range [IQR]: 55 to 71) and the study included 74 (58%) women. The majority had a metastatic lesion in the lower extremity (78% [100/128]). Eighty (63%) patients had previous surgery, and 72 (56%) had previous radiotherapy (Table 1). Breast was the most common primary tumor type (26%) (Table 2).

## 3. Results

### 3.1. Patient Perceived Compared to Clinician MSTS Score

We found that the clinicians' MSTS score overestimated the physical function as compared to the patient perceived score. The median clinician MSTS score was 8 points higher (median: 65 and IQR: 49 to 83) as compared to the patient perceived score (median: 57 and IQR: 40 to 70) (*p* < 0.001) (Table 3). This difference also existed when analyzing the lower extremity and upper extremity versions separately (Table 3).

When comparing the three common domains, clinicians scored higher for function (*p* < 0.001) and emotional acceptance (*p* < 0.001) as compared to the patient perceived score; however, there was no difference in assessment of pain (*p* = 0.076). When comparing the three lower extremity specific domains, clinicians scored higher for use of supports (*p* = 0.003) and gait (*p* = 0.006) as compared to the patient perceived score, and there was no difference in assessment of walking ability (*p* = 0.102). When comparing the three upper extremity specific domains, clinicians scored higher for hand-positioning (*p* = 0.048) and lifting ability (*p* = 0.002) as compared to the patient perceived score, and there was no difference in assessment of dexterity (*p* = 0.890).

Agreement between the overall clinician score and the patient perceived score was substantial (ICC: 0.66, 95% CI 0.43–0.79, and *p* < 0.001) (Table 4). We found moderate agreement for assessment of the common domains: pain (ICC: 0.50) and function (ICC: 0.43), but no agreement for emotional acceptance (ICC: 0.08). Agreement was substantial for assessment of the lower extremity specific use of supports domain (ICC: 0.72) and moderate for walking ability (ICC: 0.47) and gait (ICC: 0.48). We found substantial agreement for the upper extremity specific hand-positioning domain (ICC: 0.61), moderate for dexterity (ICC: 0.51), and no agreement for lifting ability (ICC: 0.16). The Spearman rank correlation coefficients were higher than the intraclass correlation coefficients reflecting the discrepancy of scores between the clinician and patient (Table 4).

### 3.2. Assessing Reliability of Extracting the Clinician MSTS Score from Medical Records

We found no difference in overall clinician MSTS score derived from medical records between researchers (researcher 1: median: 67 and IQR: 48–90 and researcher 2: median: 63 and IQR: 50–82; *p* = 0.142), nor did we find a difference between researchers for deriving any of the medical record based domain scores. The interobserver agreement between researchers for the overall clinician MSTS score was substantial (ICC: 0.78, 95% CI 0.70–0.84, and *p* < 0.001). These analyses indicate substantial reliability for deriving the clinician MSTS score from the medical record.

## 4. Discussion

The MSTS scoring tool evaluates function in patients with extremity tumors and is developed to be completed by the clinician [3]. It is unclear how this clinician-based score relates to the patients perceived function. We therefore compared the MSTS score as completed by the patient with a medical record based clinician reported MSTS score and assessed discrepancies and agreement. We found that the clinicians' MSTS score overestimated physical function as compared to the patient completed MSTS score. This discrepancy was the largest for the common overall function and emotional acceptance domains but was absent for the pain domain.

This study has limitations. First, we based the MSTS score on review of information provided by the clinician in the medical records; however, the MSTS score was developed to be completed by a clinician at time of the consultation. We see this as an important limitation and explored its possible consequences by assessing discrepancies and interobserver agreement between two researchers who independently derived these data from medical records. There was no discrepancy between the researchers for the overall MSTS score and their interobserver agreement was substantial; this suggests reproducible assessment of the MSTS score based on the medical record. Previous studies used the same methodology to extract an MSTS score from information in the medical record [15–17]. In addition, the judgment of the two research fellows might have been different from the judgment of the attending surgeon. Future prospective study should therefore compare the patient completed MSTS score with an MSTS score completed by the clinician at time of the consultation. Second, patients might have misunderstood specific items or answer options as the scoring system is not developed to be completed by a patient and not validated in a patient sample. We considered this as a limitation but feel that this did not compromise our results, as we believe that erroneous answers would have occurred in both directions (i.e., better and worse). Third, the MSTS score is developed for evaluation of functional status in all musculoskeletal tumor types. Patient demographics differ per tumor type and we only studied a sample of patients with bone metastases; this limits the generalizability of our results to this specific population. Future study might help elucidate the discrepancy between patient and physician perceived function in primary bone tumors.

Previous studies in other fields also demonstrated an overestimation of patients' physical and mental health when estimated by a clinician as compared to the patients' perception [10, 13, 18]. Nelson et al. [10] demonstrated in 1,101 primary care patients that 12% rated major physical limitations in the preceding month, while only 4.4% of the patients were rated as such by their primary care physician. This study also demonstrated that 9% rated major emotional limitations, while only 5% were rated as such by their physician. Rosenberger et al. [18] demonstrated that physicians overestimated function and underestimated pain in 98 patients who underwent surgical anterior cruciate ligament reconstruction or meniscectomy. In line with these previous studies, we found the largest discrepancy for assessment of the function and emotional acceptance domains in our study. However, we found no difference for the pain domain. Pain level in the MSTS score is based on the amount of pain and the degree of disability it causes; this might explain why we did not find difference in pain score. Despite the discrepancies, clinicians' estimates do correlate reasonably well with patient scores for the overall MSTS score and domain scores, except for emotional acceptance and lifting ability. This means that clinicians recognize worse overall function as perceived by the patient; however, the clinician tends to underestimate its impact. Assessment of emotional acceptance by the clinician does not correlate with the patients' perception, which might be explained by the subjectivity and complexity of this measure. Lifting ability is a relatively objective measure and the absence of correlation between the patient and clinician score might have been a result of the small sample size (28 upper extremity patients).

The discrepancy between the clinicians' assessment and patients perception of health and symptoms can have several consequences. First, surgeons have an important role in counseling their patients regarding expected outcome after treatment. It is important for them to understand patients' perspectives about outcome to educate future patients. For example, patients might be less satisfied, if their expectations are not met or when recovery is slower than expected [18]. Second, patients might feel misunderstood or unheard by their physician. A previous study demonstrated that concordance (so called dyadic agreement) between the patients' and physicians' perceptions of health and symptoms are associated with higher patient satisfaction [19]. Another study demonstrated that dissatisfaction of the patient leads to less compliance with treatment recommendations and potentially jeopardizes patients' health and outcome [20]. A review of plaintiff depositions demonstrated that delivering information poorly and failure to empathize with the patients' or family's perspective are common causes of medical litigation [21, 22]. Third, a clinician might be biased towards certain treatments; this might compromise comparison of clinician reported outcomes across treatment options in prospective studies and nonblinded clinical trials. Fourth, overestimating outcomes tends to breed an attitude of complacency and inertia among clinicians which could preclude further improvement. Fifth, third-party payers may use reported (overestimated) outcomes to dissuade costly innovation and research.

Capturing patient reported outcome measures, questionnaires completed by the patient, using validated instruments for both research purposes and day-to-day clinical practice is key. Previous studies demonstrated that use of information from patient reported outcome measures leads to better communication and decision making between doctors and patients and improves satisfaction [11, 23, 24]. However, this does not mean that clinician measures are uninformative. Measuring pathophysiology and impairment (e.g., range of motion, strength, and stability), in addition to patient reported outcome measures (e.g., symptoms and disability), will help us to better understand patient perceptions and inform them about prognosis and outcome of different treatment options.

In conclusion, clinician reports overestimate function as compared to the patient perceived score. This is important to acknowledge when informing patients about the expected outcome of treatment and to understand patients' perceptions. Our study reinforces the need for obtaining patient reported outcomes using validated methods in orthopaedic oncology.

## Figures and Tables

**Table 1:** Demographics (*n* = 128).

	Median (±interquartile range)
*Age *	63 (55–71)

	*n* (%)

*Sex*	
Women	74 (58)
Men	54 (42)

*Race*	
Caucasian	117 (91)
African American	10 (8)
Asian	1 (1)

*Education*	
High-school or less	41 (32)
College or Bachelor's degree	53 (41)
Graduate or professional degree	34 (27)

*Marital status *	
Married	84 (66)
Single	15 (12)
Widowed	15 (12)
Separated/divorced	9 (7)
Living with partner	5 (4)

*Location of metastasis*	
Upper extremity	28 (22)
Humerus	21 (16)
Scapula	5 (4)
Clavicule	1 (1)
Radius	1 (1)
Lower extremity	100 (78)
Femur	71 (55)
Acetabulum	14 (11)
Pelvis	12 (9)
Tibia	2 (2)
Fibula	1 (1)

*Other disabling conditions*	
Yes	37 (29)
No	90 (71)

*Previous surgery for metastatic lesion*	
Yes	80 (63)
No	48 (38)

*Previous radiotherapy for metastatic lesion*	
Yes	72 (56)
No	53 (41)
Unknown	3 (2)

*Multiple bones affected*	
Yes	61 (48)
No	55 (43)
Unknown	12 (9)

*Visceral organs affected*	
Yes	39 (30)
No	78 (61)
Unknown	11 (9)

**Table 2:** Tumor type (*n* = 128).

*Bone metastases*	
Breast	33 (26)
Renal cell	17 (13)
Prostate	11 (8.6)
Lung	11 (8.6)
Melanoma	7 (5.5)
Leiomyosarcoma	5 (3.9)
Bladder	3 (2.3)
Thyroid	3 (2.3)
Colorectal	2 (1.6)
Hepatocellular	2 (1.6)
Stomach	1 (0.8)
Esophageal	1 (0.8)
Neuroendocrine	1 (0.8)
Sarcoma	1 (0.8)

*Primary bone tumors*	
Myeloma	17 (13)
Lymphoma	13 (10)

**Table 3:** MSTS score comparison.

	Patient score	Clinician score	*p* value
	Median (±interquartile range)	Median (±interquartile range)	
*Overall MSTS score *	57 (40–70)	65 (49–83)	**<0.001**
Lower extremity MSTS score	57 (37–70)	63 (48–84)	**<0.001**
Upper extremity MSTS score	63 (53–73)	74 (58–85)	**<0.001**

*MSTS common domains*			
Pain	4 (2–5)	3 (3-4)	0.076
Function	2 (2–4)	4 (3–5)	**<0.001**
Emotional acceptance	2 (1–3)	4 (3-4)	**<0.001**

*Lower extremity specific domains*			
Use of supports	1 (1–5)	3 (1–5)	**0.003**
Walking ability	4 (3-4)	3 (3-4)	0.102
Gait	3 (2–4)	4 (3–5)	**0.006**

*Upper extremity specific domains*			
Hand-positioning	4 (1–5)	4 (3-4)	**0.048**
Dexterity	5 (3–5)	4 (4-5)	0.890
Lifting ability	3 (1–4)	4 (3-4)	**0.002**

Bold indicates significant difference (two-tailed *p* value below 0.05).

**Table 4:** Comparison of interobserver reliability.

	Patient score compared with clinician score	*p* value	Patient score compared with clinician score	*p* value
	Spearman correlation coefficient (95% confidence interval)^*∗*^		Intraclass correlation coefficient (95% confidence interval)	
*Overall MSTS score*	0.74 (0.64–0.83)	**<0.001**	0.66 (0.43–0.79)	**<0.001**
Lower extremity MSTS score	0.71 (0.59–0.82)	**<0.001**	0.64 (0.42–0.78)	**<0.001**
Upper extremity MSTS score	0.82 (0.68–0.97)	**<0.001**	0.74 (0.25–0.90)	**<0.001**

*MSTS common domains*				
Pain	0.50 (0.35–0.64)	**<0.001**	0.50 (0.36–0.62)	**<0.001**
Function	0.52 (0.38–0.66)	**<0.001**	0.43 (0.23–0.59)	**<0.001**
Emotional acceptance	0.16 (−0.02–0.35)	0.073	0.08 (−0.06–0.21)	0.105

*Lower extremity specific domains*				
Use of supports	0.74 (0.62–0.86)	**<0.001**	0.72 (0.60–0.81)	**<0.001**
Walking ability	0.54 (0.39–0.68)	**<0.001**	0.47 (0.30–0.61)	**<0.001**
Gait	0.49 (0.34–0.63)	**<0.001**	0.48 (0.29–0.62)	**<0.001**

*Upper extremity specific domains*				
Hand-positioning	0.84 (0.72–0.96)	**<0.001**	0.61 (0.29–0.81)	**<0.001**
Dexterity	0.51 (0.18–0.85)	**0.003**	0.51 (0.18–0.74)	**0.003**
Lifting ability	0.30 (−0.07–0.66)	0.110	0.16 (−0.12–0.46)	0.124

Bold indicates significant correlation (two-tailed *p* value below 0.05).

^*∗*^95% confidence interval calculated through bootstrapping (1,000 resamples).
